# Influences of lumbo-sacral transitional vertebrae for anterior lumbar interbody fusion

**DOI:** 10.1038/s41598-024-53179-w

**Published:** 2024-02-02

**Authors:** Luis Becker, Tim Victor Mihalache, Hendrik Schmidt, Michael Putzier, Matthias Pumberger, Friederike Schömig

**Affiliations:** 1 Centrum für Muskuloskeletale Chirurgie, Charité-Universitätsmedizin Berlin, Charitéplatz 1, 10117 Berlin, Germany; 2 Berlin Institute of Health, Julius Wolff Institute for Biomechanics and Musculoskeletal Regeneration, Charité-Universitätsmedizin Berlin, Augustenburger Pl. 1, 13353 Berlin, Germany

**Keywords:** Musculoskeletal system, Bone, Skeleton

## Abstract

Lumbo-sacral transitional vertebrae (LSTV) are frequent congenital variances of the spine and are associated with increased spinal degeneration. Nevertheless, there is a lack of data whether bony alterations associated with LSTV result in reduced segmental restoration of lordosis when performing ALIF. 58 patients with monosegmental stand-alone ALIF in the spinal segment between the 24th and 25th vertebra (L5/S1)/(L5/L6) where included. Of these, 17 patients had LSTV and were matched to a control population by age and sex. Pelvic incidence, pelvic tilt, sagittal vertical axis, lumbar lordosis, segmental lordosis, disc height and depth were compared. LSTV-patients had a significantly reduced segmental lordosis L4/5 (p = 0.028) and L5/S1/(L5/L6) (p = 0.041) preoperatively. ALIF resulted in a significant increase in segmental lordosis L5/S1 (p < 0.001). Postoperatively, the preoperatively reduced segmental lordosis was no longer significantly different in segments L4/5 (p = 0.349) and L5/S1/(L5/6) (p = 0.576). ALIF is associated with a significant increase in segmental lordosis in the treated segment even in patients with LSTV. Therefore, ALIF is a sufficient intervention for restoring the segmental lordosis in these patients as well.

## Introduction

The reported prevalence of lumbosacral transitional vertebrae (LSTV) ranges from 5 to 36% in the literature^1,2^. LSTV tend to be reported at higher prevalences in cohorts of patients with back pain and appear to vary regionally^3^. However, the relationship between LSTV and back pain as well as an increased incidence of spinal degeneration is discussed controversially^3–5^. A segmentally reduced mobility in the transitional segments and compensatory increased mobility in other segments is discussed as a potential cause for the predisposition of patients with LSTV to spinal degeneration^3,6^. In the context of Bertolotti’s syndrome, LSTV have been associated to back pain for nearly a century. In Bertolotti’s syndrome, enlargement of the transverse process results in a pseudarticulation of the transverse process with the os sacrum or bony fusion, resulting in lateral nerve compression or lower lumbar pain in the region of the pseudarthrosis^1^.

Treatment options for Bertolotti’s syndrome are primarily conservative with analgesia, physical therapy, infiltration therapy, or radiofrequency ablation of the joint. In the absence of improvement of symptoms, partial resection of the enlarged transverse process has been reported with heterogeneous results or lumbar fusion is discussed^7^.

The literature shows that in the context of a lumbar fusion, the segmental restoration of the lordosis is associated with a reduction of the load on the adjacent segments and thus a risk reduction of adjacent segment degeneration^8^. With regard to global spinal alignment, restoration of the sagittal profile with balanced lumbar lordosis relative to pelvic incidence (PI) and concordance between the C7 plumb-line and the base of the os sacrum appear to be associated with better patient outcome^8^. Thereby, restoration of the segmental lordosis angle in the context of monosegmental fusions is better achieved by retroperitoneal than by dorsal procedures^9,10^. However, the impact of single level fusion on global alignment is controversial in the literature^10–12^.

Determining the most appropriate approach in patients with LSTV is challenged by the concomitant changes in soft tissue anatomy that accompany the changes in bony anatomy^13^. To date, there is a lack of data on whether an increased bony connection between the lowest lumbar vertebral body and the sacral bone due to enlargement or bony fusion of the transverse process with the os sacrum in patients with LSTV has an influence on the radiological restoration of the segmental lordosis angle and the global sagittal alignment.

## Methods

### Study population

**Figure 1:**
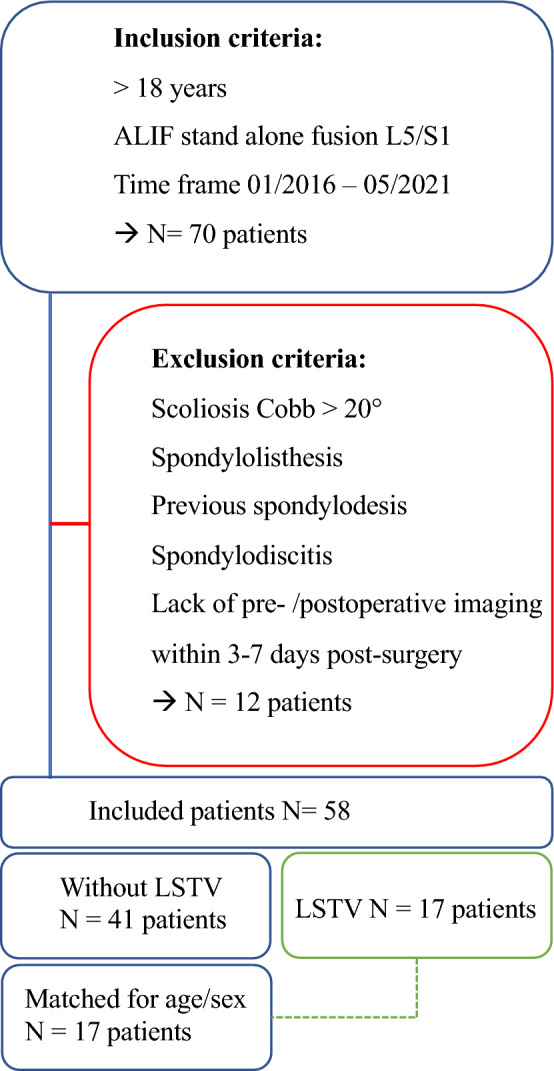
Flowchart of patient inclusion.

A retrospective matched-pair analysis using pre- and postoperative full spinal radiographs for ALIF surgery that was approved by the ethics board of Charité University Berlin (EA4/155/21) was performed. Written informed consent was waived by the ethics board of Charité University Berlin due to the retrospective study design. The study was performed in accordance with the declaration of Helsinki and followed the STROBE checklist. Patients older than 18 years who underwent monosegmental anterior lumbar interbody fusion (ALIF) without pedicle screw instrumentation of the 24th with 25th vertebra (L5/S1 or L5/6) counting caudally from C1 in our center from 01/2016 to 05/2021 were included. Indication for fusion surgery was chronic back pain associated with osteochondrosis and disc degeneration in the lowest spinal segment with movement capacity. Exclusion criteria were scoliosis with a Cobb angle > 20°, spondylolisthesis, previous spinal spondylodesis, suspected spinal infection, and missing pre- and postoperative full spinal X-rays in standing position between days 3 and 7 postoperatively. Seventy patients met the inclusion criteria. Eleven patients had to be excluded due to lack of timely pre- or postoperative imaging, while one patient was excluded due to scoliosis, resulting in a final inclusion of 58 patients. Of these, 17 patients had LSTV. These were matched for age and sex with a control population without LSTV 1:1. Inclusion flow chart is given in Fig. 1.

### ALIF procedure

**Figure 2:**
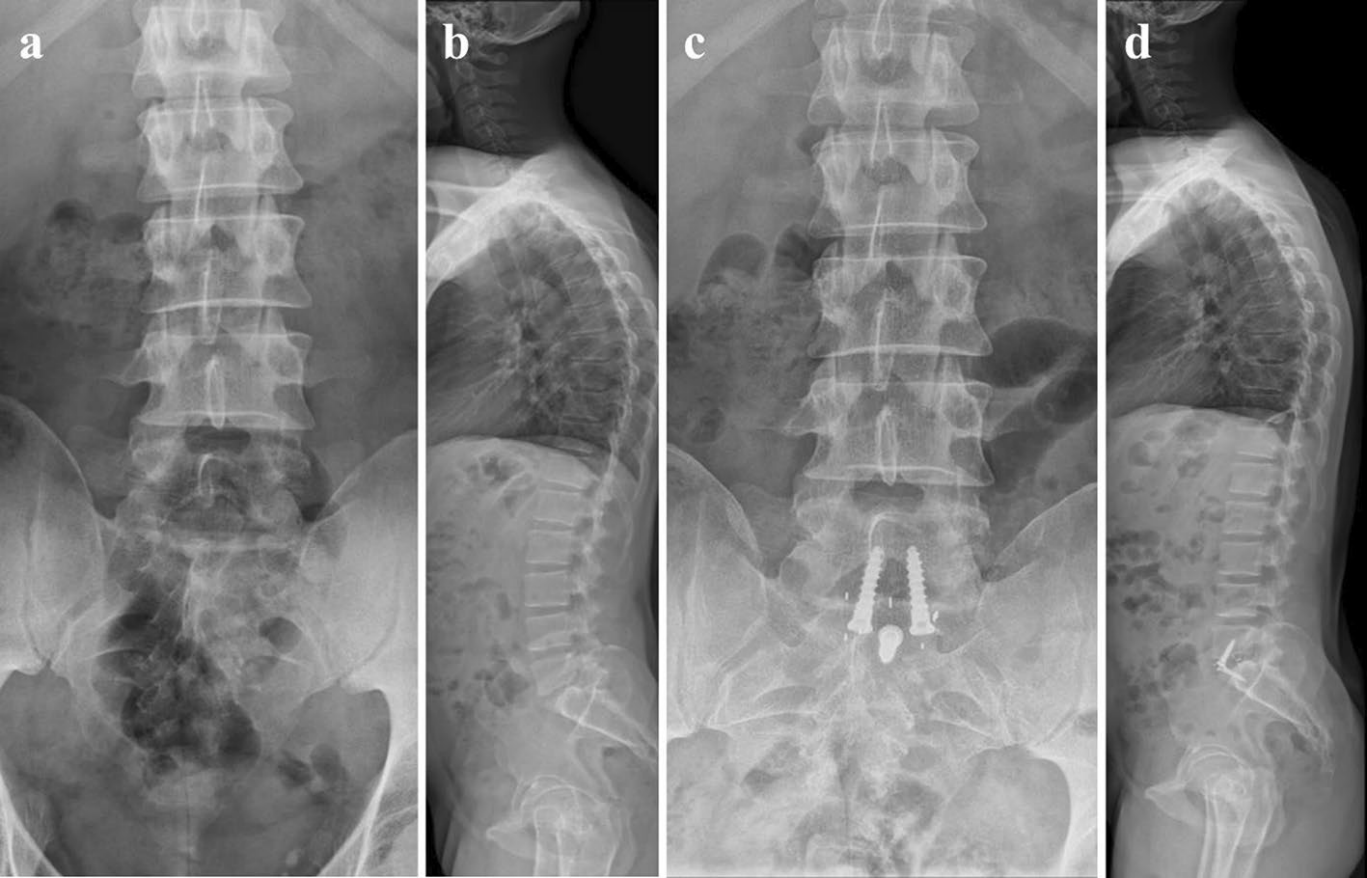
Case example. Pre- and postoperative radiographs of a 43-year-old patient with Castellvi grade Ib. (**a**,**b**) The patient’s preoperative radiographs while (**c**,**d**) depict radiographs taken after ALIF was performed.

All surgeries were performed by the senior attending surgeons (M.P. and M.P.). A left paramedian skin incision was made in the lower abdomen, followed by a sharp incision of the rectus abdominis muscle fascia. The M. rectus abdominis was bluntly dissected laterally and entered retroperitoneally below the linea arcuata. Thereafter, blunt dissection to the sacral promontory was performed, followed by retraction of the peritoneum with the viscera to the medial side as well as the left iliac vessels to the lateral side for adequate approach to the most caudal moving segment between the 24th and 25th vertebra. Sharp incision of the longitudinal anterior ligament was performed prior to complete discectomy. Subsequently, fluoroscopy-controlled cage insertion with position control was performed as shown in Fig. 2.

### Classification

**Figure 3:**
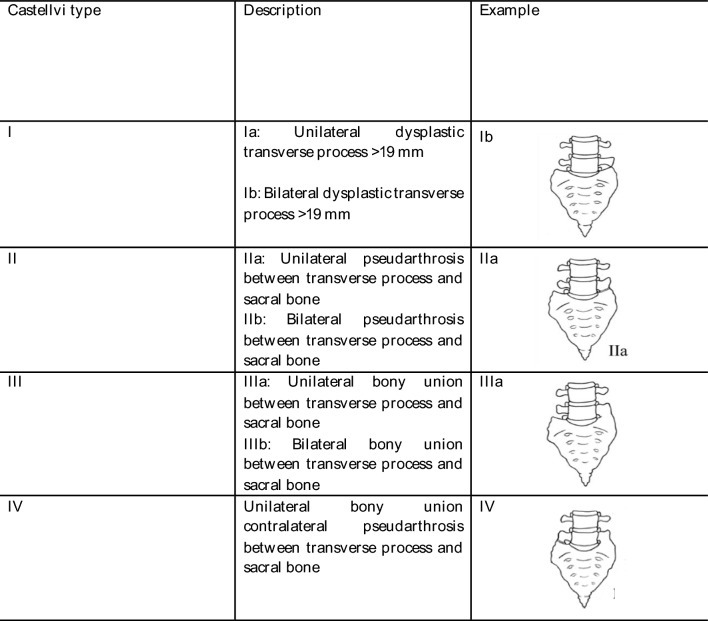
Castellvi classification.

Osteochondrosis was classified according to the Modic classification^14^. Disc degeneration was evaluated according to the Pfirrmann classification^15^. LSTV were classified according to Castellvi by an orthopedic resident with 3 years of experience after by an attending spine surgeon with 10 years of experience. An enlarged transverse process was classified as Castellvi I, pseudarthrosis of the enlarged transvers process with the os sacrum as Castellvi II, unilateral or bilateral osseous fusion as Castellvi III, and unilateral fusion with contralateral pseudarthrosis as Castellvi IV as presented in Fig. 3^16^. Vertebral bodies were classified by counting caudally from C1 in whole-spine images. For the cervical spine, seven vertebrae were assumed, and twelve for the thoracic spine. L1 was defined as the 20th vertebra. Sacralization was defined if the 24th vertebra, lumbarization if the 25th vertebra presented transition according to Castellvi. As every participant in our collective with lumbarization exhibited at least Castellvi ≥ 3 and therefore bony fusion between the 25th and 26th vertebra, no movement capacity between the 25th and 26th vertebra was assumed. All measurements were performed using Phönix PACS software (Phönix PACS GmbH, Freiburg im Breisgau, Germany).

### Measurements

**Figure 4:**
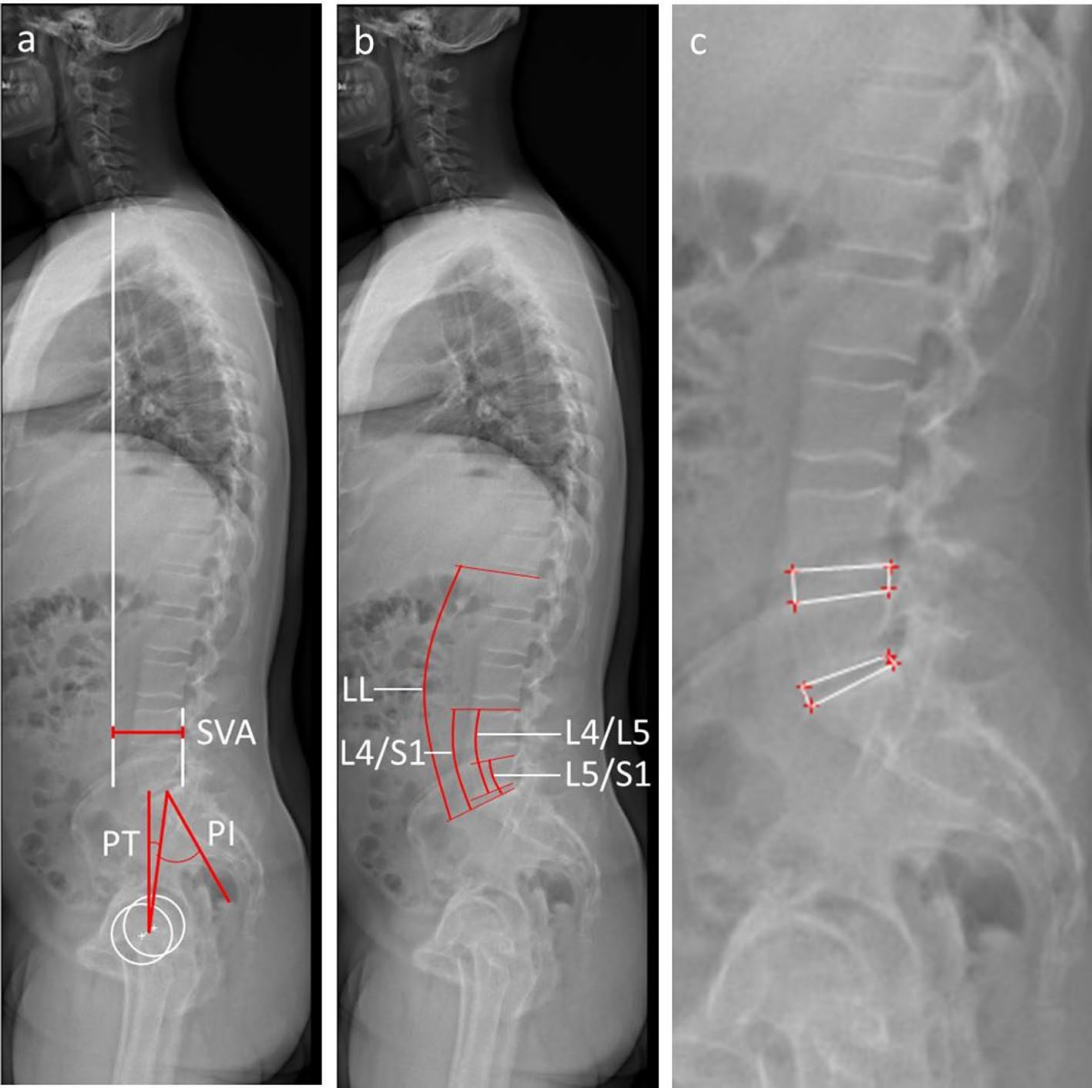
Radiologic measurements. (**a**) The measurement of the sagittal vertical axis (SVA) as the distance of the line between the plumb line of the midpoint of the body of the 7th cervical vertebra and the plumb line of the S1 dorsal superior apex. Also shown in (**a**) is the measurement of pelvic incidence (PI) as the angle of the S1 upper endplate orthogonal and a line connecting the center of the S1 upper endplate and the center of the femoral heads. The center of rotation of the femoral heads was defined as the midpoint of the line passing through both femoral head centers. Pelvic tilt (PT) was defined as the angle between a line connecting the center of the S1 upper endplate and the axis of rotation of the femoral heads and the vertical perpendicular of the center of rotation of the femoral heads. (**b**) The determination of lumbar lordosis (LL) as the Cobb angle from the upper end-plate L1 to upper end-plate of S1. The bisegmental lordosis of L4/S1 was determined from the upper end-plate of S1 to the upper end-plate of L4. The segmental lordosis of L5/S1 was determined from the upper endplate of S1 to the upper endplate of L5. The segmental lordosis of L4/5 was determined from the upper endplate of L4 to the lower endplate of L5. (**c**) The measurement of the cranial and caudal disc depth as well as the anterior and posterior disc height for the calculation of the disc height index.

PI and pelvic tilt (PT) were assessed using lateral whole spine X-rays, which included imaging down to the femoral heads. For patients in whom the femoral head was not congruently superimposed, the midpoint between the two femoral head centers was determined and used as the reference point for the measurements as shown in Fig. 4a.

The sagittal vertical axis (SVA) was defined as the horizontal distance between the plumb line from the midpoint of the C7 vertebral body to the posterior superior end of the endplate of the 25th vertebrae (Fig. 4a).

We determined the patients' lumbar lordosis (LL) as the angle of the 20th (L1) upper endplate to the 25th vertebra upper endplate in the lateral spinal image and the segmental lordosis angles L5/S1 between the upper endplate of the 24th (L5) and the upper endplate of 25th vertebra, the segmental lordosis angle L4/5 between the upper endplate of the 23rd (L4) and the lower endplate of the 24th vertebra (L5). The bisegmental lordosis angle L4/S1 was measured between the upper endplate of the 23rd (L4) as well as the upper endplate of the 25th vertebrae (Fig. 4b).

For the evaluation of disc alterations, the disc-height-index was calculated by measuring the height of the disc space at the anterior and posterior ends of the vertebral bodies and the cranial and caudal disc depths in lateral spine X-rays as shown in Fig. 4c. Disc-height-index (DHI) was calculated using Eq. (1)

1 $${\text{DHI}}=([\mathrm{Disc \,\,height \,\,anterior}+{\text{posterior}}]/[\mathrm{Disc \,\,depth \,\,cranial}+{\text{caudal}}])\times 100.$$

### Statistics

Statistical analysis was performed using SPSS Version 27 (IBM Corporation, New York, USA). The Kolmogorov–Smirnov test was used to test the data for normal distribution. For the statistical analysis of parametric data paired after matching, the paired t-test was used. For nonparametric paired data, the Wilcoxon-rank-sum test was used. For testing correlations, Pearson's correlation coefficient was used for normally distributed data and Spearman's correlation coefficient was used for data not following normal distribution. The intraclass coefficient was evaluated to assess inter-rater reliability. The significance level was set at p < 0.05 for all tests.

## Results

### Patients

A total of 58 patients was included, 32 of which were female. Mean age was 47.6 years. Of the included patients, three had Castellvi grade I, ten had Castellvi grade II, three had Castellvi grade III, and one had Castellvi grade IV. In the cohort of patients with LSTV, lumbarization of the first sacral vertebra was observed in four cases, sacralization of the last lumbar vertebra in 13 cases. The matched groups did not differ significantly in age (p = 0.687) or sex (p = 1.000). Used cages for ALIF procedure between matched groups as well as Modic classification and disc degeneration according to Pfirrmann are given in Tables 1 and 2.

**Table 1:** ALIF Cages implanted.

	Cages	
	LSTV (n = 17)	Control (n = 17)
Size		
32 × 23 mm (n)	9	10
37 × 27 mm (n)	8	7
Height		
10 mm (n)	0	1
12 mm (n)	3	1
14 mm (n)	14	15
Lordosis		
8° (n)	2	3
12° (n)	15	14

**Table 2:** Osteochondrosis according to Modic and disc degeneration according to Pfirrmann in the matched groups.

	Degeneration	
	LSTV (n = 17)	Control (n = 17)
Osteochondrosis (Modic)		
I° (n)	4	1
II° (n)	13	15
III° (n)	0	1
Disc degeneration (Pfirrmann)		
I° (n)	0	0
II° (n)	0	0
III° (n)	1	1
IV° (n)	9	8
V° (n)	7	8

### Influence of ALIF on radiologic parameters

**Figure 5:**
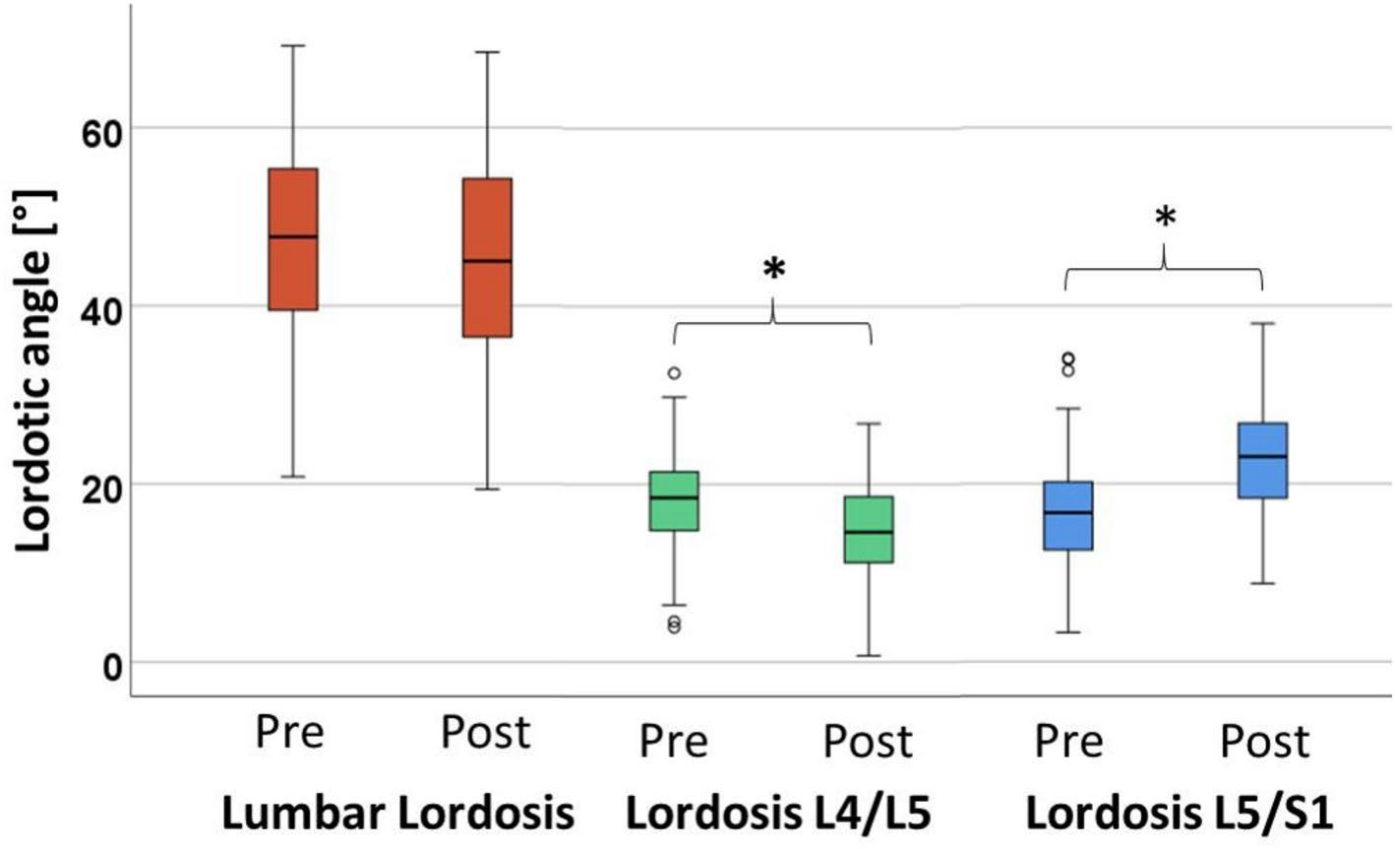
Lordotic angle for global lumbar lordosis and segmental lordosis L4/5 and L5/S1. This figure presents the preoperative and postoperative values for lumbar lordosis and segmental lordosis L4/5 and L5/S1 in our cohort of 58 patients. Significant pre- to postoperative differences are marked by asterisks.

Interrater reliability presented excellent agreement with Cronbachs Alpha value of 0.958 for sagittal spinal alignment parameters. In the L5/S1 segment, ALIF resulted in a significant increase (p < 0.001) in segmental lordosis from a preoperative median of 16.8° (7.9°) to 23.1° (8.5°) postoperatively as shown in Fig. 5. The L4/5 segment showed significantly reduced lordosis after ALIF (p < 0.001) from 18.5° (6.8°) to 14.6° (8.0°). Looking at the lower lumbar section L4-S1, ALIF led to a significant increase in lordosis from 28.4° (9.0°) to 33.7° (10.5°) (p < 0.001). However, the increased lordosis in the lower lumbar spine did not lead to an increased total lumbar lordosis (p = 0.186).

ALIF did not influence SVA (p = 0.885) or the mismatch between pelvic incidence and lumbarlordosis mismatch (PI-LL mismatch) (p = 0.253) significantly. Decreased preoperative lumbar lordosis correlated weakly but significantly with greater sagittal imbalance (p = 0.017, r = − 0.314). Similarly, postoperatively decreased lumbar lordosis correlated significantly with an increase in postoperative sagittal imbalance (p = 0.011, r = − 0.331).

The increased lordosis in the L5/S1 segment was accompanied by a significant increase in PT (p = 0.023) with a PT from preoperative 11.8° (8.5°) to postoperative 12.7° (7.7°) whereas the fixed spino-pelvic parameter PI showed no significant changes (p = 0.514) (Table 3).

**Table 3:** Impact of ALIF on sagittal alignment.

	Preoperative median (± IQR)	Postoperative median (± IQR)	p value
SVA (mm)	35.9 (40.5)	29.6 (44.6)	0.885
PI (°)	45.5 (14.2)	46.9 (12.8)	0.514
PT (°)	11.8 (8.5)	12.7 (7.7)	**0.023**
LL (°)	47.8 (16.1)	45.0 (18.4)	0.186
PI-LL mismatch (°)	6.7 (8.2)	7.7 (11.2)	0.253
SGL L4/L5 (°)	18.5 (6.8)	14.6 (8.0)	** < 0.001**
SGL L5/S1 (°)	16.8 (7.9)	23.1 (8.5)	** < 0.001**
DHI L5/S1	20.6 (7.1)	35.0 (9.8)	** < 0.001**

This table gives the pre- and postoperative radiological parameters in the cohort of 58 patients. Significant differences are marked in bold. *SVA* sagittal vertical axis, *PI* pelvic incidence, *PT* pelvic tilt, *LL* lumbar lordosis, *PI-LL* *mismatch* pelvic incidence-lumbar lordosis mismatch, *SGL L4/5* segmental lordosis L4/5, *SGL L5/S1* segmental lordosis L5/S1, *DHI L5/S1* disc height index L5/S1, interquartile range* IQR*. Due to normal distribution of SVA, PT, LL, SGL L4/5, SGL L5/S1, DHI L5/S1 a paired t-test was used for statistical comparison. PI did not follow normal distribution, which is why Wilxocon rank sum test was used.

There was a significant increase in intervertebral disc space height after discectomy and cage application (p < 0.001) for the L5/S1 segment. Likewise, there was an increase in disc height L1/2 (p = 0.015), L2/3 (p = 0.010), L3/4 (p < 0.001), while no significant changes were observed for the L4/5 segment (p = 0.091).

The observed postoperative effects were confirmed in the follow-up of more than 3 months in all 15 patients (6 LSTV, 9 control), who received follow-up radiographs in our department. Here, patients showed no significant changes for lumbar lordosis, segmental lordosis L4/5 or segmental lordosis L5/S1 even in the longer term compared to the short-term postoperative outcome as shown in Table 4.

**Table 4:** Postoperative short-term and follow-up radiographic outcome.

	Postoperative median (± IQR)	Follow-up median (± IQR)	p value
LL (°)	44.3 (24.7)	45.3 (17.5)	0.277
SGL L4/L5 (°)	13.9 (5.2)	15.3 (5.9)	0.050
SGL L5/S1 (°)	22.0 (6.2)	21.2 (3.2)	0.287

This table gives the postoperative and follow-up radiological outcome in 15 patients, for which radiographic follow-up of more than 3 months was available. Median follow-up time was 10 months (span 3–55 months). *LL* lumbar lordosis, *SGL L4/5* segmental lordosis L4/5, *SGL L5/S1* segmental lordosis L5/S1. Due to normal distribution of LL and SGL L4/5 a paired t-test was used for statistical comparison. SG L5/S1 did not follow normal distribution, which is why Wilcoxon rank sum test was used.

### ALIF in patients with LSTV

There was no significant difference in the PI pre- (p = 0.605) or postoperatively (p = 0.796) between patients with LSTV and the control group. Similarly, there were no significant differences in pelvic tilt pre- (p = 0.959) or postoperatively (p = 0.654) between patients with LSTV and the control group.

Patients with LSTV showed no significant differences in disc height compared to the matched control collective neither pre- (p = 0.332) nor postoperatively (p = 0.981). Patients with LSTV and the control collective showed a comparable increase in height of the L5/S1 disc space after ALIF (p = 0.702). Similarly, patients with LSTV and the control collective did not differ significantly in the height of the discs in the L1–L5 segments neither pre- (L1/2 p = 0.271, L2/3 p = 0.992, L3/4 p = 0.781, L4/5 p = 0.688) nor postoperatively (L1/2 p = 0.514, L2/3 p = 0.667, L3/4 p = 0.099, L4/5 p = 0.964).

Patients with LSTV showed significantly reduced segmental lordosis in both the L4/5 (p = 0.028) and the L5/S1 (p = 0.041) segments preoperatively with no significant difference in overall lumbar lordosis (p = 0.748) compared to the control group. For the pre- to postoperative change in segmental lordosis in the L4/5 segment (p = 0.116) as well as the surgically addressed L5/S1 segment (p = 0.170), patients with LSTV showed no significant differences compared to the control group. Similarly, patients with LSTV did not differ significantly (p = 0.908) from the control collective with respect to changes in the total lumbar lordosis due to ALIF. There were no significant differences in the segmental lordosis of the L4/5 (p = 0.349) or L5/S1 (p = 0.576) segments or the total lumbar lordosis (p = 0.714) between patients with LSTV and the control collective postoperatively. Differences between patients with LSTV and the control group are presented in Table 5.

**Table 5:** Segmental and lumbar lordosis for patients with LSTV and control group.

	Preoperative			Postoperative		
	LSTV Median (IQR)	Control Median (IQR)	p value	LSTV Median (IQR)	Control Median (IQR)	p value
SVA (mm)	43.6 (42.2)	43.6 (39.3)	0.886	33.2 (42.9)	53.3 (51.4)	0.419
PI (°)	44.4 (14.7)	43.8 (14.7)	0.605	46.0 (10.8)	42.7 (13.6)	0.796
PT (°)	12.0 (9.4)	10.9 (5.0)	0.959	12.6 (7.9)	11.9 (6.3)	0.654
LL (°)	44.4 (12.4)	50.9 (21.0)	0.748	43.2 (18.5)	49.5 (13.2)	0.714
PI-LL (°)	-0.9 (15.0)	-0.1 (12.9)	0.159	4.3 (13.8)	1.2 (15.1)	0.741
SGL L4/L5 (°)	17.3 (7.7)	19.7 (8.9)	**0.028**	13.3 (5.7)	15.9 (8.5)	0.349
SGL L5/S1 (°)	14.3 (9.7)	19.0 (8.4)	**0.041**	19.8 (9.4)	24.2 (8.5)	0.576
DHI L5/S1	17.1 (5.4)	20.5 (4.8)	0.332	32.5 (11.4)	34.2 (7.4)	0.981

This table gives the differences between patients with LSTV and the matched control group pre- and postoperatively. Significant differences are marked in bold. *SVA* sagittal vertical axis, *PI* pelvic incidence, *PT* pelvic tilt, *LL* lumbar lordosis, *PI-LL mismatch* pelvic incidence-lumbar lordosis mismatch, *SGL L4/5* segmental lordosis L4/5, *SGL L5/S1* segmental lordosis L5/S1, *DHI L5/S1* disc-height-index L5/S1. SVA pre- and postoperative, PI preoperative, PT pre- and postoperative, LL pre- and postoperative, PI-LL mismatch pre- and postoperative, SGL L4/5 pre- and postoperative, SGL L5/S1 pre- and postoperative and DHI L5/S1 postoperative followed normal distribution, which is why a paired t-test was used for statistical analysis. PI postoperative and DHI L5/S1 did not follow normal distribution, which is why Wilcoxon rank sum test was used.

## Discussion

We investigated the influence of ALIF on segmental as well as lumbar lordosis, pelvic tilt, and global spino-pelvic alignment in patients with LSTV compared to a matched control group. Our results show that ALIF is associated with an increase in segmental lordosis for the treated segment. Preoperatively, patients with LSTV showed significantly reduced values for segmental lordosis of the treated segment as well as of the cranial adjacent segment, while after ALIF, these values no longer differed significantly between patients with LSTV and the control group. Therefore, ALIF should be considered as a sufficient intervention for restoring segmental lordosis in patients with LSTV.

In the literature, restoration of lumbar lordosis is associated with a reduction in the rate for degeneration in the adjacent segment^17^ while decreased segmental lordosis in lumbar fusion poses a risk for adjacent segmental degeneration^18^. Consistent with the literature, ALIF has a significant impact on segmental lordosis of the treated segment in our collective in both patients with and without LSTV^9,19^. According to the literature, compared to posterior procedures such as transforaminal or posterior lumbar interbody fusion, ALIF provides increased segmental lordosis^19^. The increase in segmental lordosis of 5.2° ± 6.2° on average obtained in our study is slightly lower than the values of about 6°–8° reported in the literature^10,19^. However, the achieved postoperative segmental lordosis after ALIF in the last mobile lumbar segment of 22.5° ± 5.9° is within the range of the literature^20^. In our study, ALIF had no significant effect on overall lumbar lordosis or SVA. The influence of ALIF on postoperative overall lumbar lordosis is also discussed controversially in the literature. While some authors report a significant increase in lumbar lordosis^9,10^, others show no significant changes in postoperative lordosis after ALIF^21–23^. A study by Tung et al. even shows a reduction in lordosis directly postoperatively after ALIF, but the lordosis increased back to the preoperative baseline at the 2-year follow-up^21^. Kim et al. show no significant change in lordosis shortly postoperatively, but a significant increase at 24 months follow-up^20^. The reduced mobility and loss of disc height in segment L5/S1 preoperatively results in reduced segmental lordosis, which may be compensated by increased lordosis of adjacent segments to maintain sagittal balance^24^. The increased segmental lordosis in the treated segment due to ALIF resulted in a reduction of the segmental lordosis of the cranial adjacent segment L4/5 in our study. This might result from a compensatory hyperlordosis in the adjacent segment preoperatively, which postoperatively is no longer needed for maintenance of sagittal balance due to the increased segmental lordosis at the treated segment^24^. In line with the results previously reported by Lightsey et al., patients did not show a significant reduction of the achieved segmental lordosis in follow-up examinations after ALIF compared to the early postoperative outcome^25,26^. While these results are based on a small number of patients, they show a trend towards achieving a significant lordosis in the treated segment in the long term by performing ALIF.

SVA is considered a relevant parameter for the postoperative outcome as well as the risk for reoperations due to adjacent segment degenerations^26^. Patients with reduced lumbar lordosis had an increased risk for sagittal imbalance in our study. To date, there is ongoing controversy in the literature regarding the effect of ALIF on the global spinal alignment in terms of a change in SVA^21,22,27^. Consistent with the findings of Boissiere et al. and Afathi et al., ALIF did not show a significant effect on SVA in our collective^12,22^.

Pelvic tilt is a significant factor influencing clinical outcome with an increased PT and associated pelvic retroversion correlating with a worse outcome regarding health questionnaires such as the Oswestry Disability Index^28^. The effect of ALIF on PT is, however, controversial in the literature. While Boissiere et al. and Kim et al. report no significant effect^20,22^, Hosseini et al. and Janjua et al. present a significant reduction of PT by ALIF^28,29^. Marouby et al. show an increase in PT, which was not statistically significant but was comparable to our results in which we observed a slight increase in PT^27^. This may be explained by the relatively low PT in our cohort compared with the literature. A review by Formica et al. showed that the mean PT in the studies they examined averaged 17.4 ± 4.2, whereas our study had a mean postoperative PT of only 13.5° ± 7.5°^30^.

Patients with LSTV showed no significant differences in pelvic configuration or pre- and postoperative PT compared to the control group. These results are in contrast to findings of Haffer et al. who detected significantly increased PT in patients with LSTV^31^. This may be due to their inclusion of a larger cohort of patients with higher-graded transitional vertebrae and a positive correlation between the degree of transition of LSTV and PI in their collective. In our study, patients with LSTV showed no significant difference in PT compared to a control group. The literature gives controversial evidence for the relationship between pelvic tilt and LSTV. Belindayi et al. describe a significantly reduced sacral tilt in patients with LSTV^32^, whereas Yokoyama et al. and Price et al. found an increased PT for patients with LSTV^33,34^. These differences may be influenced because the results were derived based on the Castellvi classification, which does not distinguish between lumbarization and sacralization. However, Mahato demonstrated that through changes in facet and auricular surface^35^, sacralization and lumbarization can be seen as distinct entities, which emphasizes the understanding of the biomechanical characteristics of LSTV and associated back pain^36^. Patients with LSTV in our cohort showed a significantly reduced preoperative segmental lordosis of L4/5 as well as the last mobile segment L5/S1 (L5/L6) compared to the control group. In patients with LSTV, despite increased bony contact due to transitional vertebrae and soft tissue alterations such as musculare^13^, ligamentous^37^ and vascular adaptions^38^ which might have an influence on tension and biomechanics of the treated segment^39^, sufficient lordosis of the last mobile segment was achieved by ALIF, causing a postoperative compensation of preoperative differences. Similarly, patients with LSTV showed a reduced lumbar lordosis preoperatively, possibly due to the reduced segmental lordosis in the last mobile segment, which was compensated to the level of the control group postoperatively due to the increased segmental lordosis in the treated segment.

Some limitations need to be discussed. First, the classification of LSTV was based on standard anterior–posterior radiographs while Ferguson radiographs are usually considered standard radiographic imaging and CT is most sensitive in detecting LSTV. This may have caused a falsely low classification of LSTV. The relatively small population of 17 patients per group in the matched cohorts can possibly be seen as a drawback of the study. However, to the authors' knowledge, only one study by Weiner et al. from 2001 exists to date, which retrospectively analyses 12 patients after ALIF regarding vascular anatomy^40^. There is no data describing the influence of ALIF on the sagittal profile of patients with LSTV. Our results show that ALIF also leads to sufficient re-lordosing in patients with LSTV and thus expand the treatment options for patients with LSTV. Furthermore, in the postoperative follow-up, an influence of increased muscle tone due to pain on the sagittal profile may have resulted. This was, however, reduced by the application of adequate standardized analgesia protocols. As the study was performed retrospectively, long-term follow-up radiographs were only available from a low number of patients as were standardized clinical parameters. Thus, statistical effects might have been biased. However, to our knowledge, this still is the largest analysis of ALIF in LSTV patients with a matched control group.

In conclusion, performing ALIF is associated with a significant increase in segmental lordosis in the segment being treated in both patients with and without LSTV. Preoperatively, patients with LSTV show significantly reduced values for segmental lordosis compared to the control group. Postoperatively, patients with LSTV no longer show significant differences in segmental lordosis or lumbar lordosis. Thus, ALIF is a sufficient intervention for restoring segmental lordosis and disc-height even in patients with LSTV.

## Data Availability

The authors confirm that the data supporting the findings of this study are available within the article.
